# Neural Mechanisms of Reciprocity Availability and Expectancy Violation During Social Interaction

**DOI:** 10.3390/brainsci16020222

**Published:** 2026-02-13

**Authors:** Daniele Olivo, Andrea Di Ciano, Lucia Giudetti, Riccardo Cazzaro, Fabio Sambataro

**Affiliations:** 1 Department of Neuroscience (DNS), University of Padova, 35121 Padova, Italy; 2 Padova Neuroscience Center, University of Padova, 35129 Padova, Italy; 3 Fondazione Giancarlo Quarta, 20129 Milan, Italy

**Keywords:** social cognition, reciprocity, fMRI, functional connectivity, expectancy violation

## Abstract

**Background**: Reciprocity is a core mechanism of social bonding, signaling whether others are available and willing to provide support. The perception of reciprocity availability fosters trust and belonging, whereas its absence may elicit expectancy violation and negative affect. This study investigated the neural correlates of reciprocity availability (RA) and unavailability (RU) during social interaction. **Methods**: Thirty healthy adults underwent a social task during a functional magnetic resonance imaging (fMRI) scan while viewing short vignettes depicting social exchanges differing in reciprocity cues. Univariate and multivariate (MVPA) analyses were used to identify activation and connectivity patterns associated with RA and RU. Affective responses, reaction times, and personality traits were correlated with neural activity. **Results**: RA engaged the ventromedial prefrontal cortex, precuneus, temporoparietal junction, and visual cortices. RU elicited greater activation of the left inferior frontal gyrus, dorsomedial prefrontal cortex, and temporal pole, along with enhanced connectivity between visual and parieto-temporal regions. In exploratory analyses, agreeableness correlated with ventromedial prefrontal activation during RA, whereas depressive temperament correlated with temporal pole activity during RU. **Conclusions**: Reciprocity availability versus unavailability engages distinct large-scale networks for socio-emotional integration and expectancy monitoring, defining a mechanistic framework for studying disrupted reciprocity in psychopathology.

## 1. Introduction

Evolutionary accounts converge on the idea that belonging and inclusion have adaptive value for survival; accordingly, forming social relationships and affiliating with groups fulfill a fundamental human need [1]. Maintaining supportive relationships, however, is a dynamic and complex process grounded in trust and reciprocity, which shape how individuals perceive and respond to others’ availability [2].

A substantial body of work has documented the mental-health benefits of social connection and the deleterious effects of isolation and loneliness [3,4]. By contrast, fewer studies have examined the neural architecture of reciprocity—the core mechanism underpinning cooperative exchange and social bonding. Reciprocity is commonly described as a give-and-take process that reflects the balance of exchanges between individuals; although it does not imply perfectly equal returns, it highlights an inherent drive to cooperate, whether rooted in altruism or self-interest [5]. Beyond tangible exchanges, reciprocity conveys signals of relational availability—that is, subtle cues that another individual is emotionally and behaviorally attuned to one’s needs. From this perspective, reciprocity operates not only through overt acts but also through implicit communicative cues that inform expectations about another’s willingness to sustain cooperative engagement. Such cues reduce social uncertainty and promote security, enduring connection, and belonging [6]. Conversely, when reciprocity cues are absent or ambiguous, individuals may experience expectancy violation, often accompanied by negative affect such as frustration or rejection [7,8].

Functional magnetic resonance imaging (fMRI) has become central to social neuroscience, revealing how the brain encodes complex interpersonal contingencies, including inclusion/exclusion dynamics and social expectations [9]. Neural systems supporting social cognition include the so-called “social cognition network” [10], particularly the medial prefrontal cortex (MPFC), temporoparietal junction (TPJ), and posterior cingulate cortex (PCC) which contribute to representing others’ intentions and emotional states and to maintaining a sense of relational continuity [11,12]. Affective processing regions such as the amygdala and insula may also contribute, especially when interpreting emotional signals that reinforce relational stability. The engagement of these regions during perceived continuity can support mental representation of the partner, integration of prior interactions, and anticipation of future exchanges, thereby fostering relational resilience [13,14,15].

Experimental paradigms examining reciprocity-related behavior typically involve cooperative exchange settings (e.g., helping, trust, or fairness contexts) and converge on the idea that reciprocal responses carry motivational value and promote a sense of interpersonal safety [16]. From a neurobiological perspective, reciprocity availability is often linked to engagement of valuation and reward-related systems, including the salience system (involving the ventrolateral prefrontal cortex—VLPFC), the fronto-parietal/executive control network (involving the dorsolateral prefrontal cortex—DLPFC), the default mode network (involving the ventromedial prefrontal cortex—VMPFC), and the sensorimotor network (involving the supplementary motor area—SMA), consistent with the computation of subjective value in social contexts, mentalizing processes, and motor/embodied components supporting reciprocal behavior [17,18]. Conversely, reciprocity unavailability may elicit expectancy violation and enhanced recruitment of regions involved in social inference, cognitive appraisal, and integration of negative interpersonal meaning [19,20]. Together, this evidence supports the conceptualization of reciprocity not merely as a behavioral exchange rule, but as a cue signaling relational availability that shapes expectations and affective responses during ongoing interactions.

Building on this literature, the present study investigates how cues of reciprocity availability (RA) versus reciprocity unavailability (RU) modulate affective and neural responses during social interaction. Using short, ecologically framed vignettes of everyday situations, we experimentally manipulated the presence or absence of communicative signals indicating a partner’s relational availability [21]. Although participants were familiarized with the characters before scanning to enhance ecological validity, the manipulation targeted momentary reciprocity cues within a single interaction, not the longitudinal continuity of real relationships.

Our prior work using a well-established social interaction paradigm showed that (1) benefiting from helping behaviors engages regions implicated in mentalizing/theory of mind—including bilateral superior temporal sulcus (STS), TPJ, temporal pole (TP), and MPFC—and that (2) exposure to socially aversive interactions is associated with greater right occipital–temporal activation, reduced homotopic functional connectivity of temporal cortex, and hyperactivation of dorsomedial and lateral prefrontal regions relative to prosocial interactions [22]. Extending this line of research, we hypothesized that perceived unavailability of reciprocity (RU) would preferentially recruit fronto-temporal regions associated with expectancy violation and socio-emotional conflict, whereas reciprocity availability (RA) would engage networks implicated in affiliative reward, self-referential processing, and social bonding.

To test these hypotheses, we combined univariate analyses with multivariate pattern analysis (MVPA) to characterize activation and connectivity patterns distinguishing RA and RU. Given the established link between negative affect and the perceived unavailability of relational support, we further examined whether these neural responses were associated with individual differences in personality traits. Understanding how the brain encodes immediate reciprocity cues may illuminate the mechanisms supporting trust, cooperation, and emotion regulation in everyday social exchanges.

## 2. Materials and Methods

### 2.1. Participants

Thirty right-handed native Italian speakers (16 males, 14 females; age range = 20–30 years; mean age ± SD = 23.8 ± 2.1) with normal or corrected-to-normal vision participated in the study. Participants’ education level ranged from 13 to 18 years (mean ± SD = 14.8 ± 1.6). Exclusion criteria included: (1) any history of substance abuse or dependence (excluding nicotine); (2) head trauma with loss of consciousness; (3) current medical or neurological illness; (4) lifetime psychiatric disorders; (5) documented intellectual impairment; (6) contraindications to MRI; and (7) excessive head motion during scanning. Participants were screened for current and past psychiatric disorders through a standardized medical history and a clinician screening interview focusing on major psychiatric conditions (mood, anxiety, psychotic, and substance-use disorders). Exclusion criteria included any self-reported history of psychiatric diagnosis, as well as current psychological/psychiatric treatment. Any medication intake was assessed at enrollment, and participants taking any medication were excluded.

All participants provided written informed consent prior to the study. The experimental procedures were approved by the Ethics Committee of the University of Parma (protocol 552/2020/SPER/UNIPR) and conducted in accordance with the Declaration of Helsinki.

According to standard estimates for within-subject fMRI designs, a sample of N = 30 provides >80% power to detect medium effect sizes (Cohen’s d = 0.6) at α = 0.05 FWE-corrected. This a priori estimate was based on general recommendations for within-subject fMRI designs [23] rather than on a single prior effect size from an identical paradigm. The chosen medium effect size (d ≈ 0.6) was intended to reflect typical within-subject activation differences observed in task-fMRI contrasts and to provide a pragmatic target given feasibility constraints.

### 2.2. Social Interaction Task

**Figure 1:**
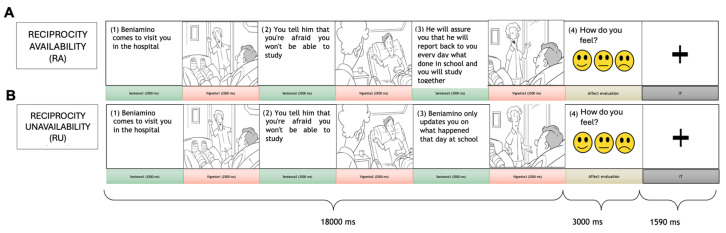
The social interaction task. Each trial included two parts: the display of a social interaction scene composed of three sentences alternated with three vignettes and an affect rating response. The intertrial interval was presented in between trials. The participant was asked to identify himself/herself with the gray-haired person, the receiver, who interacted with another person, and the agent. Two forms of social interaction were presented: RA conditions (**A**), and RU condition (**B**).

Participants were shown a sequence of three pictures alternated with three sentences, depicting a dyadic social interaction (Figure 1). To increase the ecological validity of the study and promote a minimal sense of familiarity with the characters, participants were introduced one week before scanning to three different social contexts (“in the hospital with legs in plaster”; “the three childhood friends”; “colleagues”). They were asked to identify with a specific character—the recipient—who interacts with an agent. Before entering the scanner, participants were briefly retested to ensure correct identification of and recall for the contexts. The retest consisted of a short structured procedure in which participants were asked to (i) identify each context and associated characters, and (ii) provide a brief free-recall summary of the scenario. When uncertainties emerged, standardized clarifications were provided by the experimenter to ensure that all participants entered the scanner with a correct understanding of the contextual background. This step was intended to minimize potential confounds related to misunderstanding of the narrative context and to ensure that condition effects were driven by reciprocity cues rather than by contextual biases.

This preparatory step aimed to facilitate emotional engagement and a baseline sense of reciprocity, from which the experimental manipulation could elicit condition-specific neural responses.

Each vignette illustrated a brief social exchange between the two characters. The intervening text slides provided short descriptive statements of comparable length and syntactic complexity across all conditions, helping participants to maintain a coherent mental representation of the interaction. The paradigm comprised two within-subject conditions: Reciprocity Availability (RA) and Reciprocity Unavailability (RU). In the RA condition, the agent responded to the recipient’s implicit need or request with verbal or behavioral cues of relational availability and willingness to provide help or support. In the RU condition, the agent neglected or dismissed these implicit cues, conveying unavailability or disinterest in maintaining mutual support. These manipulations targeted the perception of reciprocity cues within a single, momentary social interaction rather than long-term relationship continuity. The visual complexity, contextual balance, and linguistic content of the vignettes were carefully matched across conditions. A total of four runs each with 30 non-repeating trials were presented in a pseudo-randomized sequence identical for all participants. This procedure was adopted to minimize habituation effects and to reduce potential order-related confounds, while maintaining a comparable distribution of conditions and contexts across the four runs. In addition, using the same run order for all participants ensured a fully standardized administration of the task across subjects. No more than two trials of the same condition appeared consecutively. Each trial consisted of three 3500 ms sentence slides alternated with three 2500 ms vignette slides (total duration = 18 s), followed by a 3 s affective evaluation phase during which participants selected one of three labels (positive, neutral, or negative). This brief categorical evaluation served primarily as a manipulation check to verify that the vignettes elicited the expected affective valence and to maintain participants’ attention throughout the session, rather than as a continuous psychometric measure of emotional intensity. A fixation cross was presented for 1590 ms between trials. All visual stimuli were developed in-house by the research team in collaboration with a professional illustrator, following paradigms previously validated in our laboratory [22,24]. To further control for potential confounds, we quantitatively assessed visual and linguistic stimulus properties across conditions: (i) visual complexity of the pictorial stimuli was estimated using a GIF-compression index based on the Lempel–Ziv–Welch lossless compression algorithm, computed as the ratio between the size of the compressed and original file; (ii) linguistic properties were assessed using word count and character count to verify that they did not differ between conditions.

Before data collection, a qualitative pretest with 10 external volunteers assessed the clarity, realism, and emotional coherence of each vignette; feedback from this pilot phase was used to refine ambiguous items and ensure consistency across contexts.

### 2.3. Image Acquisition

All MRI images were acquired on a 3T scanner (GE Discovery MR750, Local Health Unit of Parma). For each subject, structural T1-weighted images were acquired by a 3D-MPRAGE sequence with the following parameters: TR = 9700 ms, TE = 3.97 ms, FOV = 256 × 256 mm^2^, and voxel size = 0.5 × 0.5 × 1 mm^3^. fMRI scan was performed using a gradient-echo-planar imaging (GE-EPI) sequence with the following parameters: TR = 2000 ms, TE = 30 ms, flip angle = 90°, FOV = 240 × 240 mm^2^, and voxel size = 3.2 × 3.2 × 3.5 mm^3^. The task consisted of four runs (342 volumes each; TR = 2 s), corresponding to ~11.4 min per run and ~45.6 min total acquisition time (excluding short breaks between runs).

### 2.4. Imaging Pre-Processing

The preprocessing pipeline was executed using DPABI [25] and SPM12 [26]. The scans were visually inspected by FS and DO to exclude artifacts or lesions. All functional and anatomical images were reoriented to the AC-PC line, realigned (using a least-squares approach and a six-parameter spatial transformation) to correct for head motion, and co-registered to the individual structural scan. Anatomical images were segmented into grey matter, white matter, and CSF tissue classes, and resampled to the Montreal Neurological Institute standard space using the MNI152 template and a voxel size of 3 × 3 × 3 mm^3^ [27]. Finally, the images were smoothed using a Gaussian kernel with a full width at half maximum of 6 mm to increase the signal-to-noise ratio and compensate for residual anatomical variation across subjects. The data were analyzed using a general linear model (GLM) with statistical parametric mapping. Each trial was modeled with three block regressors: two for the social interaction (the context which included the first two vignettes and texts and provided the background of the interaction that was identical for both conditions and the last text and vignette that was specific for each condition–trial) and one for the affect rating. Each block was modeled with a boxcar and convolved with a canonical hemodynamic response function. Six motion parameters estimated during realignment were included as nuisance covariates.

### 2.5. Imaging Activation Analysis

In the first-level analyses, whole-brain t-contrast maps between the response block of conditions were computed for each subject, and in the second one, individual Contrasts were entered into a random-effects group analysis [28] obtaining spatial maps estimated for each Contrast. For all second-level analyses, we applied a *p* < 0.05 family-wise error (FWE) correction at the cluster level with an uncorrected *p* < 0.001 at the voxel level.

### 2.6. Connectome Analysis

Using the Conn toolbox [29,30], we implemented a data-driven multivariate pattern analysis (MVPA) approach [31] to classify the core connectivity characteristics. MVPA is a data-driven method designed to capture the structure of brain connectivity patterns, which are particularly relevant in social cognitive tasks where multiple systems interact dynamically [32]. This method identifies multivariate patterns of pairwise connections between all brain voxels (voxel-to-voxel covariance) and takes into account the multivariate dependencies within the data, unlike standard univariate analyses that evaluate the effects of each voxel or cluster individually. This strategy provided a model-free identification of patterns of brain connectivity that are significantly modulated by RU and RA conditions.

Pairwise connectivity matrices between each voxel and all other voxels of the brain were calculated and summarized through a singular value decomposition (SVD). From each multivariate correlation (MCOR) maps the first 10 eigenpattern components were extracted, following the recommendation by Nieto-Castañón [31], who showed that k = 10 captures the majority of functional connectivity variance without overfitting. The resulting eigenpattern scores were entered into a second-level multivariate ANCOVA to evaluate condition effects (RA > RU and RU > RA), controlling for mean framewise displacement. Statistical significance was defined at *p* < 0.001 (voxel-wise, uncorrected) and *p* < 0.05 (cluster-level, FWE-corrected). Bonferroni adjustments were applied for the number of MVPA seed clusters identified.

Then for each significant cluster, we computed seed-based connectivity post hoc effect-size maps characterizing the pattern of condition-related differences in connectivity by calculating bivariate correlations between the signal fluctuations of each voxel in that cluster and all other voxels in the brain; these maps represent the condition-related differences in correlation strength (Fisher’s z-transformed r values) between RU and RA.

To further characterize the anatomical and functional specificity of these connectivity patterns, we generated polar plots quantifying the spatial distribution of each cluster’s connectivity profile across the seven large-scale cortical networks defined by Yeo et al. [33]. This was done by computing the number of voxels showing significant connectivity overlap between each seed cluster and each of the seven canonical networks (visual, somatomotor, dorsal attention, ventral attention, limbic, frontoparietal, and default mode). The proportion of overlap was calculated relative to the total number of significantly connected voxels, providing a normalized measure of network engagement.

### 2.7. Behavioral Analysis

The behavioral responses during the affect evaluation phase of the fMRI task were recorded and averaged for each participant and analyzed offline. We expected that scenes would trigger positive affective responses during RA and not during RU. Therefore, we compared the frequency of condition-congruent ratings (RA: “positive”; RU: “negative”) versus all other responses. In addition, reaction times (RT) during the affective evaluation phase were collected and compared across conditions. To summarize task efficiency in a single-subject behavioral index for correlation analyses, we computed a reaction-time-corrected performance score (cPerf_score) defined as: cPerf_score = accuracy/meanRT, where accuracy represents the proportion of condition-congruent ratings and meanRT is the mean reaction time (ms) for the same condition. This composite index was used to reduce the number of behavioral metrics and capture a speed–accuracy trade-off.

Additionally, participants completed the Big Five Questionnaire (BFQ) [34] and the brief version of the Temperament Evaluation of Memphis, Pisa, Paris, and San Diego—Münster (TEMPS-M) [35] to assess personality and temperament characteristics.

The BIG-5 (also known as the Five-Factor Model) [34] is a widely used model of personality that measures five broad dimensions of human behavior: (1) Openness to Experience—creativity, curiosity, and willingness to try new things; (2) Conscientiousness—organization, reliability, and a tendency to be self-disciplined; (3) Extraversion—sociability, energy, and a preference for being around others; (4) Agreeableness—compassion, cooperation, and a tendency to be empathetic; (5) Neuroticism—emotional stability, with high levels indicating greater emotional instability. The TEMPS-brief is a shorter version of the TEMPS-A [35], a questionnaire designed to assess temperamental characteristics, focusing on emotional and mood states. The TEMPS-brief measures the following dimensions: (1) Depressive Temperament—tendency to experience low mood and pessimism; (2) Manic Temperament—high energy, impulsivity, and elevated mood; (3) Irritable Temperament—tendency to experience irritability and frustration; (4) Anxious Temperament—proneness to anxiety and fearfulness; (5) Cyclothymic Temperament—instability in mood, fluctuating between depressive and manic states.

### 2.8. Brain–Behavior Correlations

From the univariate activation analyses, we extracted for each subject the first eigenvariate of the time series within an 8 mm radius sphere centered on the peak coordinates of significant clusters identified at the group level for the contrasts RU > RA and RA > RU. These subject-specific summary measures were then correlated with (i) the corrected performance index (cPerf_score), reflecting task-related behavioral performance, and (ii) the individual scores on each dimension of the Big Five Questionnaire (BFQ) and the brief-TEMPS temperament inventory.

All correlations were computed using two-tailed Pearson coefficients after assessing normality of ROI-extracted activation values and behavioral/trait measures with the Shapiro–Wilk test (all *p*’s > 0.05).

Correlations between ROI responses and behavioral, personality, and temperament measures were conducted as exploratory analyses. Across the 11 behavioral/trait variables (five Big Five dimensions, five TEMPS dimensions, and the cPerf_score) and the 10 ROI responses, a total of 110 correlations were computed. Because these tests are not statistically independent due to intercorrelations among both trait measures and ROI responses, the effective number of independent comparisons was estimated using an eigenvalue-based dimensionality-reduction approach (Li and Ji method) [36], which derives the effective number of tests (M_eff_) from the eigenvalue decomposition of the correlation matrix. This procedure yielded an adjusted significance threshold of approximately *p* < 0.001, which represents the corrected threshold accounting for multiple comparisons. Given the exploratory aim of these analyses and the limited sample size, uncorrected *p*-values are reported for descriptive purposes.

We explicitly acknowledge that given the number of tested associations across ROIs and behavioral/trait measures, correlation analyses were considered exploratory and were not used to support confirmatory inferences, serving to identify potential associations between neural activation patterns, behavioral indices, and individual personality traits to guide future studies.

## 3. Results

### 3.1. Behavioral Results

Condition-congruent affective ratings were highly frequent in both conditions: participants predominantly selected a positive label during RA trials (81.9 ± 12.0%, mean ± SD) and a negative label during RU trials (78.5 ± 13.1%, mean ± SD). Reaction times during the affective evaluation phase were significantly shorter for RA than RU (RA: 947 ± 237 ms; RU: 1110 ± 323 ms; t = −5.70, *p* < 0.001). One subject was excluded due to a response recording problem, and personality data from five subjects were unavailable.

### 3.2. Stimulus Complexity Control

No significant differences in visual complexity were observed between conditions (RA: 0.58 ± 0.06; RU: 0.57 ± 0.07; *p* = 0.536). Linguistic length of the sentence material was assessed using total word count and character count. Word count and character count did not differ significantly between conditions (word count: RA 13.07 ± 4.12 vs RU 11.50 ± 4.31, *p* = 0.156; character count: RA 70.83 ± 24.50 vs RU 61.57 ± 25.20, *p* = 0.154), supporting the comparability of stimulus features across RA and RU.

### 3.3. Brain Imaging Results

Main Effect of Social Interaction Condition

**Figure 2:**
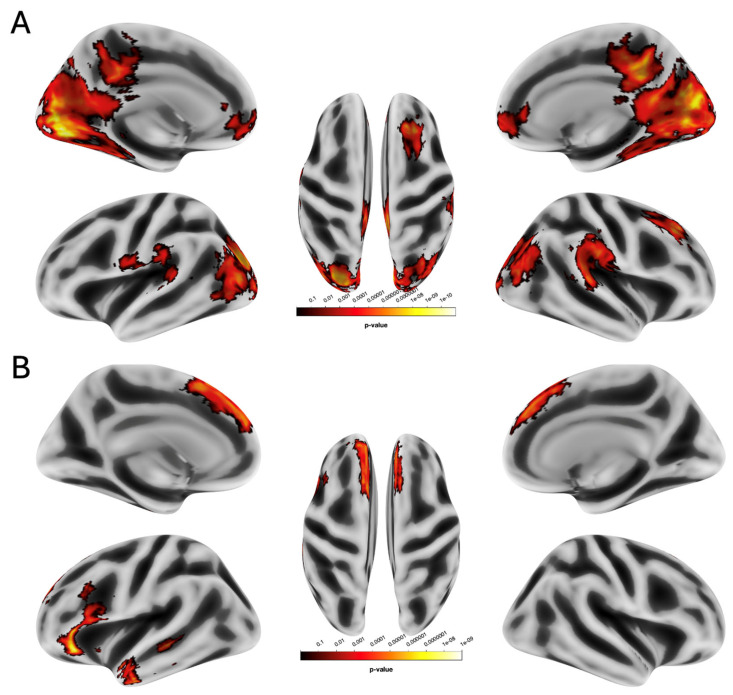
Neural effects of social interactions. (**A**) RA was associated with an increase in the calcarine cortex and lingual gyrus, bilateral ventromedial prefrontal cortex, and temporoparietal junction compared with RU. (**B**) RU was associated with increased activation in the left inferior frontal gyrus, left temporal pole, and dorsomedial prefrontal cortex in both the right and left hemispheres relative to RA. Statistical probability maps are rendered on an MNI template with a threshold of voxel-wise *p* < 0.001 and FWE-corrected *p* < 0.05 at the cluster level. MNI, Montreal Neurological Institute; FWE, family-wise error.

RA > RU: relative to RU, the RA condition showed greater activation in a large bilateral cluster of the visual cortex extending from the calcarine cortex to the lingual gyrus (CC/LG, z = 6.56, *p* < 0.001), in the right superior frontal gyrus (rSFG, z = 5.24, *p* < 0.001), bilateral ventromedial prefrontal cortex (VMPFC, z = 4.56, *p* < 0.001), and bilateral temporoparietal junction (TPJ, z = 4.00, *p* < 0.001) (see Table 1 and Figure 2A).

RU > RA: relative to RA, the RU condition showed greater activation in the left inferior frontal gyrus (left IFG, z = 5.85, *p* < 0.001), left temporal pole (leftTP, z = 5.00, *p* < 0.001), and bilateral dorsomedial prefrontal cortex (DMPFC, z = 4.73, *p* < 0.001) (see Table 1 and Figure 2B).

Multivariate pattern analysis—MVPA

**Figure 3:**
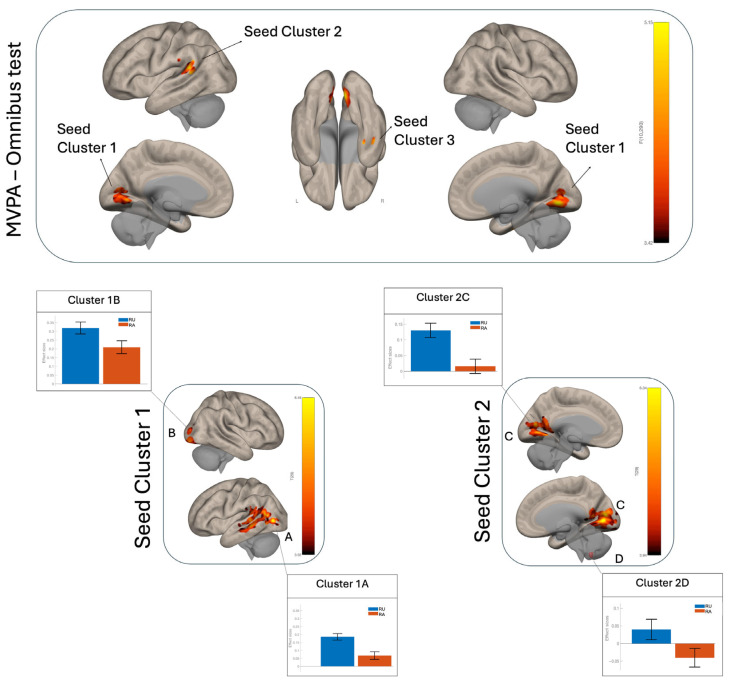
(**Top**): Multi-variate pattern analysis (MVPA) comparing RU and RA conditions. Maps were thresholded at a voxel-wise Bonferroni-adjusted level of *p* < 0.001/3 (across three MVPA-derived seeds) and a cluster-wise *p* < 0.05 FWE-corrected threshold. (**Bottom**): Seed-to-voxel post hoc connectivity maps derived from significant MVPA seed clusters. Post hoc SBC analyses were Bonferroni-adjusted across tested targets within each seed (voxel-wise *p* < 0.001/2; cluster-wise *p* < 0.05 FWE-corrected; T(29) > 3.92). Bar graphs show the mean connectivity difference (RU−RA); error bars indicate SEM.

We performed MVPA to identify condition-dependent differences in functional connectivity patterns between reciprocity availability (RA) and unavailability (RU). No significant MVPA clusters were observed for the RA > RU contrast. In contrast, the RU > RA comparison revealed three clusters whose connectivity patterns were significantly modulated by task condition, reported here in order of cluster size. Seed 1 (694 voxels) was located in the bilateral occipital cortex and cerebellum, seed 2 (168 voxels) in the left parieto-temporal cortex, and seed 3 (50 voxels) in the temporal cortex. Overall, these clusters spanned the parieto-occipital–temporal cortex (Figure 3, top). The corresponding statistical details are summarized in Table 2.

### 3.4. Post Hoc Seed-to-Voxel Results

RA > RU. No significant clusters were identified in the post hoc seed-to-voxel analysis for the RA > RU contrast.

**Figure 4:**
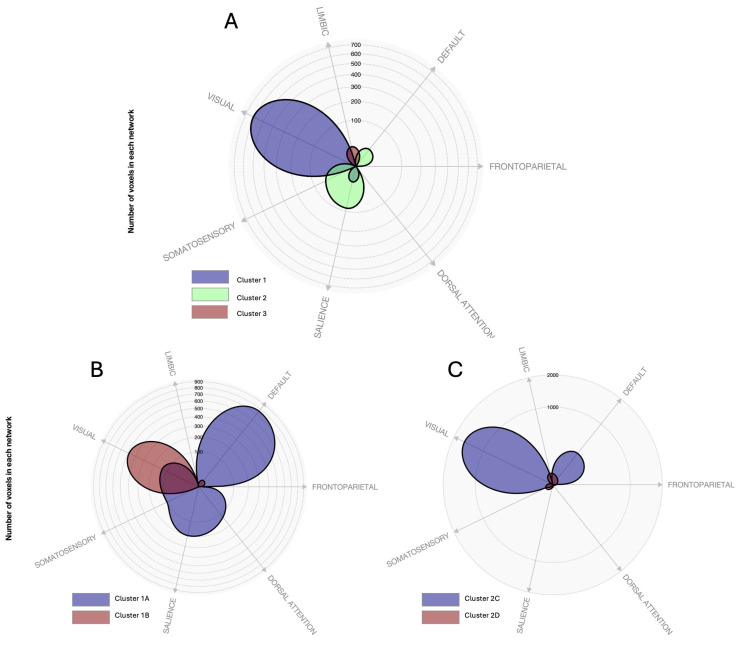
Polar display showing the number of voxels of overlap between each significant cluster of the ‘RU > RA’ comparison across different functional connectivity (FC) measures and a set of 7 canonical brain networks according to Yeo et al. [28]. (**A**) significant MVPA clusters (seed regions), (**B**) comparison of clusters 1A and 1B with canonical brain networks, and (**C**) comparison of clusters 2C and 2D with canonical brain networks. Images were created with CONN web.conn-toolbox.org (accessed on 30 August 2025).

RU > RA. In the post hoc RU > RA contrast, seed 1 showed positive functional connectivity with two clusters: cluster 1A, located in the left temporo-occipital-parietal regions mainly covering the MTG, STG, angular gyrus, supramarginal gyrus, and lateral occipital cortex, overlapping with the default mode, salience, and dorsal attention networks; and cluster 1B in the right occipital lobe, belonging to the visual network (Figure 3, left-bottom; Figure 4B). Seed 2 showed positive functional connectivity with two additional clusters: one encompassing the bilateral occipital lobes (cluster 2C), overlapping with the visual and default mode networks (Figure 4C), and one located in the right cerebellum (cluster 2D) (Figure 3, right-bottom). For seed 3, no significant clusters were identified. A detailed overlap with canonical networks is presented in Figure 4A.

### 3.5. Brain–Behavior Analysis

Correlation analyses were performed between ROI-extracted activation values, the RT-corrected performance score (cPerf_score), and personality/temperament measures (brief-TEMPS and BFQ). Normality checks using the Shapiro–Wilk test did not reveal significant deviations from normality for any variable included in the correlation analyses (all *p*’s > 0.05).

We report only those associations showing the strongest trends toward significance (*p* < 0.05, uncorrected). In the RA-related cluster (VMPFC), agreeableness was positively correlated with activation (VMPFC xyz = 3, 60, −6; r = 0.49, *p* < 0.05). In the RU-related clusters, activation in the left temporal pole (left TP; xyz = −48, −6, −36) was positively correlated with cPerf_score (r = 0.41, *p* < 0.05) and with the depressive temperament trait (r = 0.44, *p* < 0.05). In addition, the depressive temperament trait was negatively correlated with activation in the visual cortex cluster (CC/LG; xyz = −9, −81, 0; r = −0.50, *p* < 0.05), while the irritable temperament trait was positively correlated with activation in the left inferior frontal gyrus (left IFG; xyz = −51, 24, −12; r = 0.40, *p* < 0.05).

However, when accounting for the non-independence of multiple tests using the eigenvalue-based effective number of comparisons approach (see Methods), none of the observed correlations survived the adjusted exploratory significance threshold (*p* < 0.001). Accordingly, these associations are reported for descriptive purposes only.

## 4. Discussion

The present study investigated the neural and behavioral correlates of social reciprocity by manipulating the perception of another person’s relational availability during an ongoing interaction. We found that reciprocity availability (RA)—namely, the perception that a partner is willing to provide relational support—engaged the ventromedial prefrontal cortex (VMPFC), precuneus, temporoparietal junction (TPJ), and visual cortices. This pattern is consistent with the recruitment of large-scale networks implicated in socio-emotional integration, self-referential processing, and mentalizing. Conversely, reciprocity unavailability (RU)—i.e., the perception that a partner is unwilling to provide relational support—was associated with increased activation in prefronto-temporal regions, including the inferior frontal gyrus (IFG), temporal pole (TP), and dorsomedial prefrontal cortex (DMPFC), along with enhanced functional connectivity between the visual cortex and parieto-temporal regions. Taken together, these findings suggest that the brain dynamically encodes momentary reciprocity cues through partially dissociable systems that support affiliative valuation during RA and expectancy monitoring during RU.

At the behavioral level, RA vignettes elicited more positive affective evaluations and faster responses, indicating that participants reliably distinguished between reciprocity-available and reciprocity-unavailable interactions. The alignment between affective judgments and neural signatures suggests that the task successfully elicited expectancy-consistent versus expectancy-violating social scenarios. Exploratory analyses revealed descriptive trends between individual personality/temperament traits and condition-dependent neural responses (e.g., depressive temperament with TP activation during RU; agreeableness with VMPFC recruitment during RA). However, these correlations did not survive correction for multiple testing and should therefore be interpreted strictly as exploratory and hypothesis-generating [37,38]. In support of this interpretation, converging evidence indicates that the VMPFC plays a central role in value-based computations across domains, encoding social value in addition to economic value and contributing to subjective evaluation in both social and non-social contexts [39]. Similarly, affiliative and supportive interactions have been linked to the engagement of canonical reward/valuation circuitry, including the ventral striatum, reinforcing the notion that RA vignettes may be experienced as intrinsically socially rewarding [39,40].

The increased engagement of IFG and DMPFC during RU likely reflects enhanced cognitive appraisal and conflict monitoring when relational expectations are violated [41]. Both regions have been implicated in social reasoning and perspective-taking processes that become particularly relevant when individuals detect a mismatch between expected and observed reciprocity cues [42]. The temporal pole, in turn, is thought to integrate affective meaning—especially negative emotions such as sadness, anxiety, fear, and disgust—with contextual information, supporting the evaluation of socially negative events [43,44]. Consistent with this view, TP activation during RU was associated with both performance in negative-affect evaluation and depressive traits, further supporting a preferential involvement of this region in processing negatively charged interpersonal information. More broadly, RU-related effects may be conceptualized in terms of expectancy violation or social prediction error, reflecting a salient mismatch between prior relational expectations and the partner’s observed response. In line with this framework, meta-analytic evidence indicates that violations of social norms and reciprocity-related expectations consistently recruit large-scale systems supporting salience detection and cognitive control, including the anterior insula, dorsal anterior cingulate, and lateral prefrontal regions [45], and also engage networks implicated in trust and potential betrayal during reciprocal exchange [20]. Moreover, experimental studies using expectancy-incongruent social information highlight the contribution of DMPFC/TPJ to updating social inferences when others behave in ways that violate strong prior beliefs [46]. Of note, in Major Depressive Disorder (MDD), deficits in emotion regulation contribute substantially to interpersonal vulnerability, and stressful relational events such as relationship breakups may amplify stress responses and prolong distress [47]. In this context, although speculative, variability in responsivity to expectancy violations in RU-like interpersonal scenarios may represent a potential avenue for future investigation into vulnerability-related mechanisms, rather than evidence of an intermediate phenotype per se.

Importantly, several regions highlighted here—including lateral prefrontal and temporo-parietal cortices—are known to support domain-general cognitive operations such as appraisal, conceptual processing, and controlled evaluation. Thus, the RU-related pattern may reflect not only reciprocity-specific expectancy deviation but also increased cognitive elaboration and meaning construction required when participants process socially negative or ambiguous interactions. This interpretative constraint aligns with the view that reciprocity cues recruit partially overlapping systems that contribute to both social inference and general evaluative cognition.

Beyond regional activation effects, RU enhanced functional coupling between visual cortex and temporo-occipital regions within broader associative networks. Specifically, the lingual gyrus and calcarine cortex showed increased coupling with the middle and superior temporal gyri, angular and supramarginal gyri, and lateral occipital and fusiform cortices. Given the role of temporal regions in decoding socially relevant cues and negative interpersonal meaning [43,44], this connectivity pattern may reflect enhanced integration of perceptual and socio-emotional information when reciprocity cues are absent. In addition, the supramarginal, angular, and superior temporal gyri exhibited stronger connectivity with the intracalcarine–lingual gyrus, precuneus, and cerebellum—key hubs of the default mode network (DMN) and theory-of-mind (ToM) systems. Such coupling may facilitate the evaluation of expectancy violations and the simulation of potential social consequences following perceived unavailability of reciprocity. This interpretation is consistent with evidence that social expectancy deviations can elicit coordinated changes across multiple large-scale networks, including salience, default mode, and frontoparietal control networks, supporting the integration of affective alarm signaling with social inference and cognitive regulation processes.

Conversely, the engagement of VMPFC, precuneus, and TPJ during RA underscores the contribution of reward and social cognition networks to cooperative and supportive interactions. The VMPFC has been consistently linked to the subjective valuation of social reward [48,49,50], whereas the precuneus and TPJ contribute to self–other integration and perspective-taking [51]. Visual cortices also play a role in processing emotionally salient visual input and reinforcement processes during learning [52]. Within this framework, the coactivation of TPJ and VMPFC suggests a coordinated system integrating cognitive and affective information to support the maintenance of social relationships [24]. In line with prior work, interactive social contexts may simultaneously recruit mentalizing systems and reward-related circuits, highlighting the tight coupling between motivational and inferential components during ongoing social exchange [53]. RU preferentially engages systems involved in cognitive conflict, social expectancy deviation, and negative interpersonal meaning, whereas RA engages systems supporting affiliative valuation and prosocial integration.

With respect to connectivity, our MVPA results should be interpreted as reflecting condition-dependent shifts in distributed coupling configurations rather than evidence for a single, highly specific “reciprocity network.” The present study was not designed to test a priori network-level hypotheses and therefore mechanistic inferences should remain conservative. In this sense, connectivity findings are best viewed as complementary to the regional activation results and as hypothesis-generating markers of large-scale integration demands during reciprocity-unavailable scenarios.

A key consideration is that the present paradigm operationalized reciprocity availability/unavailability through brief, vignette-based scenarios rather than through fully interactive reciprocal exchanges. While this approach increases contextual richness compared with abstract economic games, it still probes perceived relational availability in a controlled and indirect manner. Accordingly, ecological validity should be interpreted as ecologically framed rather than fully naturalistic, and our results primarily inform how the brain encodes momentary reciprocity cues under constrained experimental conditions. While the present paradigm isolates momentary reciprocity cues within single interactions, future studies should extend these findings to longitudinal and dynamic designs to better capture relationship continuity. Together, these results delineate a neural framework for reciprocity processing that may inform translational investigations of psychopathological conditions characterized by disruptions in social expectation and relational attunement.

Several limitations should be acknowledged. First, the present design does not allow a complete dissociation between reciprocity availability cues and broader dimensions of prosocial support. However, the paradigm builds on previously validated vignette-based social interaction tasks demonstrating sensitivity to both prosocial and victimization contexts [22,24]. Accordingly, the findings should be interpreted as reflecting reciprocity-related relational availability signals embedded within positive versus unavailable social interaction contexts, rather than as a pure operationalization of reciprocity per se. The paradigm was not designed to provide an independent quantitative validation of perceived reciprocity availability, as its primary aim was to experimentally manipulate relational interaction contexts while controlling for visual and linguistic stimulus properties, rather than to develop or psychometrically validate a behavioral measure of reciprocity perception. Second, the sample size was relatively modest and limited to healthy young adults, which may constrain statistical power for individual-differences analyses and reduce generalizability to other age groups and clinical populations. Third, the task relied on brief vignettes depicting dyadic interactions; although this paradigm provides a more ecologically framed context, it captures momentary reciprocity availability/unavailability cues rather than fully interactive and longitudinal reciprocity dynamics. Fourth, the connectivity findings should be interpreted cautiously: given the data-driven nature of MVPA, the observed patterns are better conceptualized as condition-dependent shifts in distributed coupling configurations rather than evidence of a uniquely reciprocity-specific mechanism. Finally, when accounting for the non-independence of multiple comparisons using eigenvalue-based procedures (see Methods), none of the observed brain–behavior or brain–trait correlations survived the adjusted significance threshold; accordingly, these associations are presented solely as exploratory findings. Future studies should replicate these findings in larger samples and extend the paradigm to populations characterized by altered reciprocity processing (e.g., mood disorders, interpersonal difficulties, or social–cognitive impairments). In addition, incorporating longitudinal or interactive designs (e.g., repeated exchanges with feedback, adaptive partner behavior, or real-time social interaction) may better capture reciprocity dynamics and expectancy updating.

## 5. Conclusions

The present findings indicate that the human brain responds to perceived reciprocity availability and unavailability through partially dissociable patterns of activity across distributed prefrontal, temporal, and posterior regions. Reciprocity availability was associated with increased engagement of networks commonly implicated in mentalizing, valuation, and self-referential processing, whereas reciprocity unavailability was associated with activation in regions frequently involved in expectancy monitoring, socio-affective evaluation, and cognitive appraisal. Rather than identifying a reciprocity-specific neural mechanism, these results suggest that reciprocity-related social cues modulate the configuration of large-scale socio-affective and evaluative systems that support ongoing interpersonal interpretation. Future work in larger samples and more interactive paradigms will be needed to further clarify how individual differences and clinical conditions shape the neural processing of reciprocity-related relational signals.

## Figures and Tables

**Table 1:** Montreal Neurological Institute (MNI) coordinates of weighted centers and peaks of clusters of differences between the two conditions. RU: reciprocity unavailability, RA: reciprocity availability, left IFG: left inferior frontal gyrus, leftTP: left temporal pole, DMPFC: dorsomedial prefrontal cortex, CC: calcarine cortex, LG: lingual gyrus, rightSFG: right superior frontal gyrus, VMPFC: ventromedial prefrontal cortex, TPJ: temporoparietal junction. Cluster-size: number of cluster’s voxels. Z-score: activation peak of the cluster.

Cluster Location	Cluster-Size (Voxels)	z-Score	Peak Coordinates		
			X	Y	Z
RA > RU					
CC/LG	6730	6.56	−9	−81	0
rightSFG	194	5.24	24	33	39
VMPFC	215	4.56	3	60	−6
TPJ	137	4.00	−60	−33	21
RU > RA					
left IFG	299	5.85	−51	24	−12
leftTP	81	5.00	−48	−6	−36
DMPFC	723	4.73	−9	54	30

**Table 2:** MVPA threshold: voxel-level *p* < 0.001 (Bonferroni-adjusted across 3 contrasts), cluster-level peak p-FWE < 0.05, F(10,290) ≥ 3.39, k ≥ 50. SBC threshold: voxel-level *p* < 0.001 (Bonferroni-adjusted across 2 contrasts), cluster-level peak p-FWE < 0.05, T(29) ≥ 3.92.

Cluster Seed (MVPA)—Cluster-Size	(x, y, z)	p-FWE	Cluster Target (SBC)—Cluster-Size	(x, y, z)	p-FWE
Seed Cluster 1–694 Lingual Gyrus Right (32%), Intracalcarine Cortex Right (26%), Intracalcarine Cortex Left (18%), Lingual Gyrus Left (13%), Vermis 45 (3%), 5 Vermis (1%), Not labeled (8%)	0 −64 0	*p* < 0.01	Cluster 1A—1419 Middle Temporal Gyrus posterior division Left (19%), Angular Gyrus Left (19%), Lateral Occipital Cortex inferior division Left (12%), Middle Temporal Gyrus temporooccipital part Left (11%), Lateral Occipital Cortex superior division Left (9%), Supramarginal Gyrus posterior division Left (8%), Superior Temporal Gyrus posterior division Left (4%), Parietal Operculum Cortex Left (4%), Planum Temporal Left (2%), Not labeled (12%)	−48 −66 14	*p* < 0.01
			Cluster 1B—651 Occipital Fusiform Gyrus Right (30%), Lateral Occipital Cortex inferior division Right (27%), Occipital Pole Right (19%), Not labeled (23%)	28 −88 0	*p* < 0.05
Seed Cluster 2–168 Supramarginal gyrus posterior division left (58%), Planum Temporal Left (22%), Parietal Operculum Cortex Left (5%), Superior Temporal Gyrus posterior division Left (3%), Angular Gyrus Left (2%), Not labeled (10%)	−56 −46 18	*p* < 0.01	Cluster 2C—2199 Intracalcarine Cortex Right (26%), Precuneus Cortex (20%), Lingual Gyrus Right (12%), Intracalcarine Cortex Left (10%), Lingual Gyrus Left (9%), Occipital Pole Right (3%), Supracalcarine Cortex Right (3%), Cingulate Gyrus posterior division (1%), Supracalcarine Cortex Left (1%), Cerebellum 45 Left (1%), Not labeled (14%)	6 −72 12	*p* < 0.05
			Cluster 2D—45 Cerebellum Right (95%), Not labeled (4%)	10 −58 −54	*p* < 0.05
Seed Cluster 3—50 Temporal Fusiform Cortex (24%), posterior division Right, Inferior Temporal Gyrus (22%), posterior division Right, Not labeled (54%)	40 −20 −40	*p* < 0.001	No clusters survived		

## Data Availability

The data that support the findings of this study are available on reasonable request from the corresponding author.
